# Supplementary material for: CoGe LoadExp+: A web‐based suite that integrates next‐generation sequencing data analysis workflows and visualization
Source: Plant Direct. 2017 Jul 20;1(2):10.1002/pld3.8. doi: 10.1002/pld3.8 (PMC6508507; doi:10.1002/pld3.8)
Supplement: Supplementary file 2 [file PLD3-1-0-s002.pdf]

CoGe LoadExp+: A web-based suite that integrates next-gen sequencing data analysis workflows and visualization

Jeffrey W. Grover, Matthew Bomhoff, Sean Davey, Brian D. Gregory, Rebecca A. Mosher, and Eric Lyons

Supplementary Information

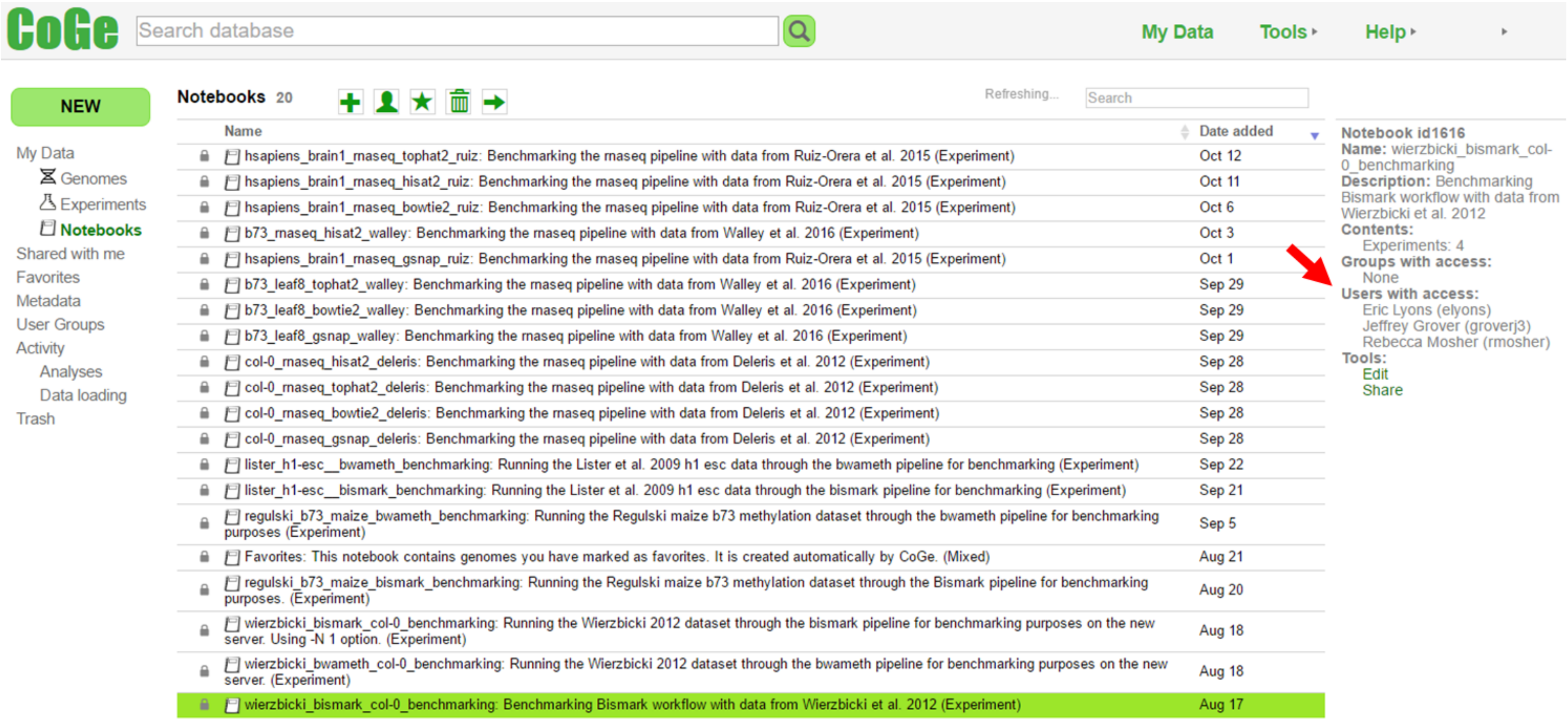

Figure S1. Data Can Be Organized Into Notebooks. Individual experiment files may be organized into one notebook. This aids in data management, allowing related datasets from LoadExp+ workflows to be grouped together and privately shared with other users or made fully public The highlighted notebook has been shared with other users (red arrow).

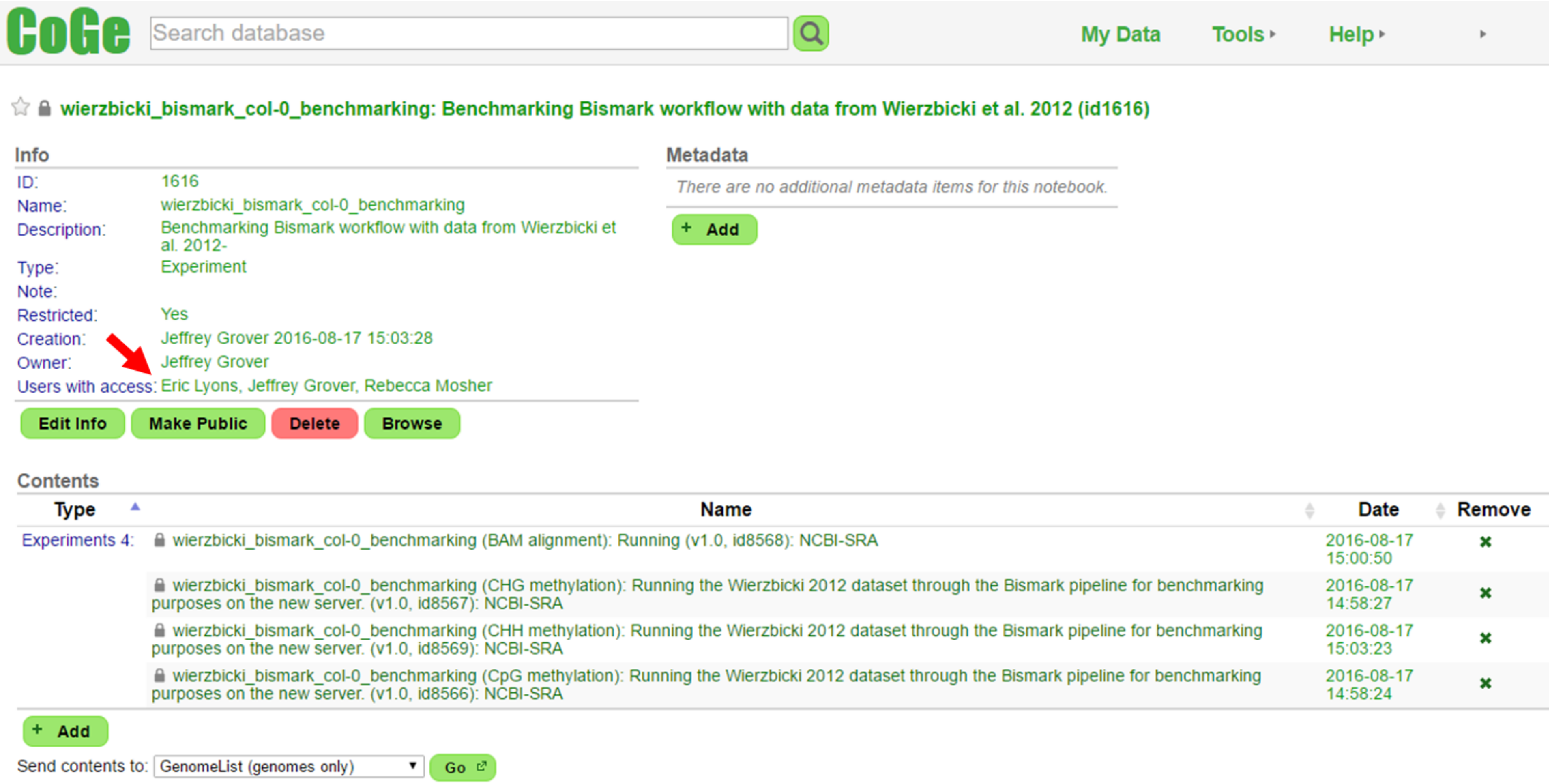

Figure S2. Notebooks Can Contain Many Experiment Files. Output files from LoadExp+ BS-seq workflows are visible inside one notebook. Clicking on the individual experiments will open them in Experiment View (Figure S4). This notebook has been shared with other users (red arrow) but otherwise is private ('Restricted: Yes').

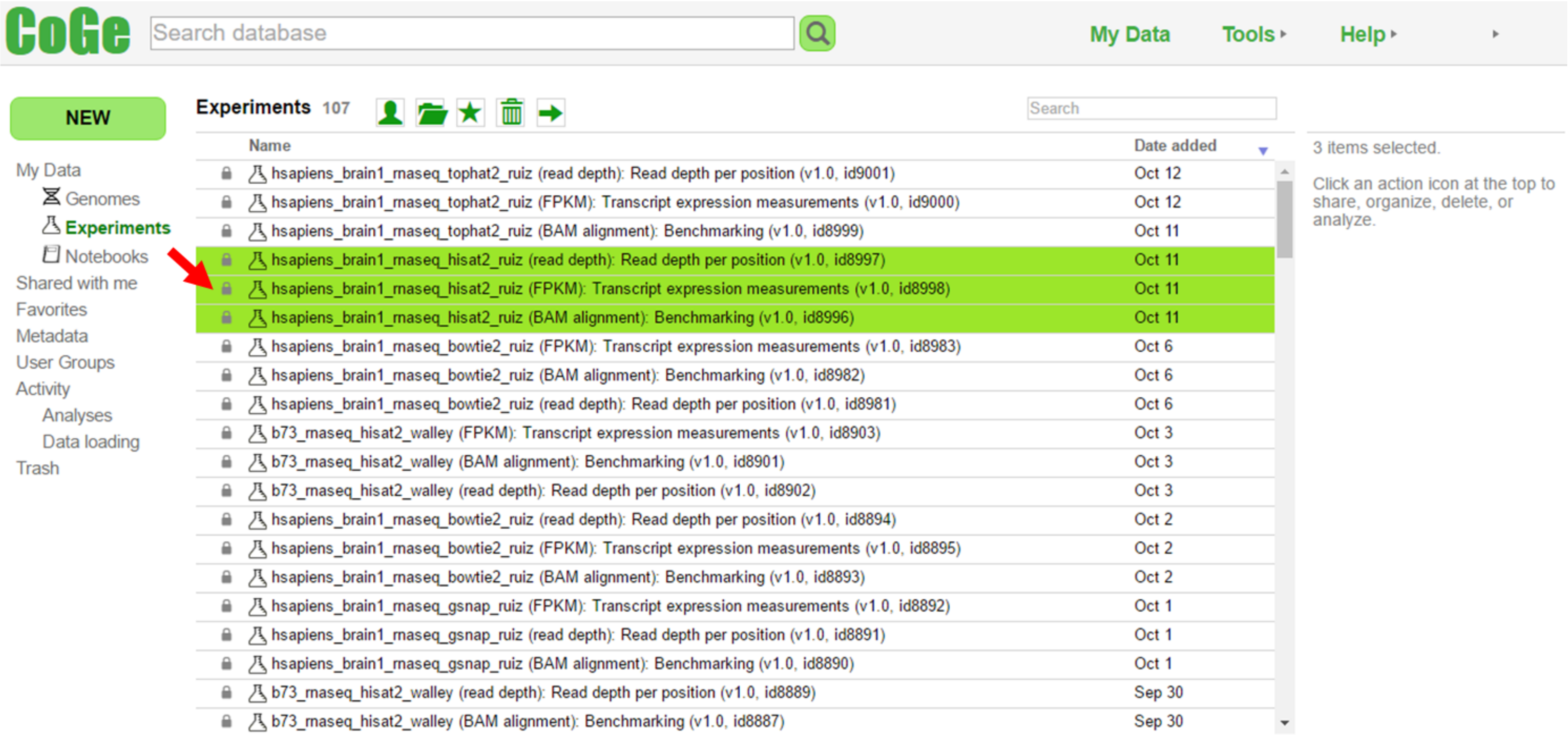

Figure S3. LoadExp+ Output Files Are Seen As Separate Experiments. Once analyses have finished a full listing of output files from any workflows that have run are visible in the user's My Data page. The three highlighted experiments (red arrow) were generated from the same LoadExp+ RNAseq run where each intermediate file of an analysis (BAM, read depth, and transcript quantification) have automatically been integrated.

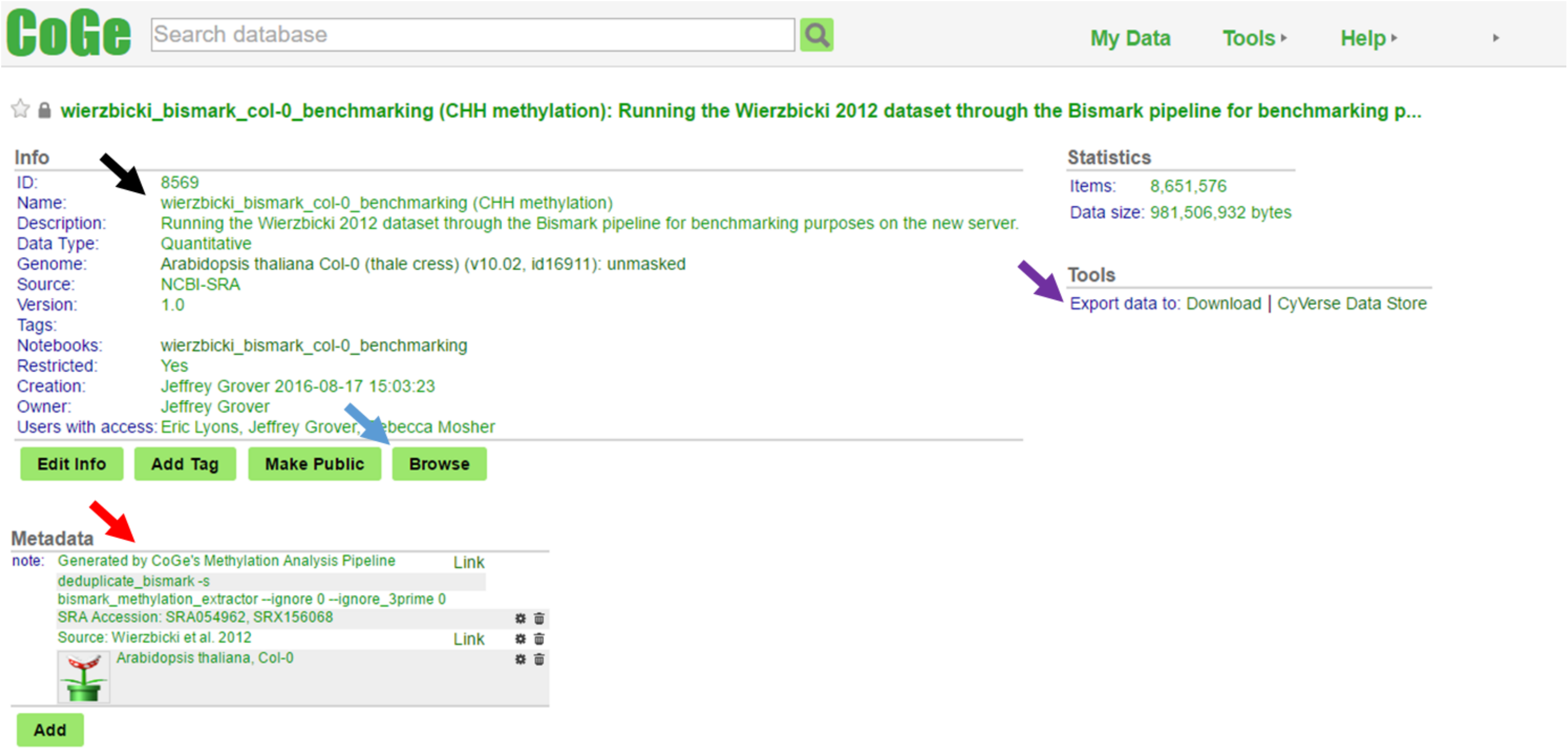

Figure S4. Experiment View. Detailed information about each output file from LoadExp+ is visible by opening that file in Experiment View. Data can also be exported for direct download or to the CyVerse Data Store (purple arrow). The experiment can be made public by clicking the "Make Public" button. User-defined metadata such as the "Name" and "Description" are visible (black arrow) and may be changed by clicking "Edit Info." Run-time options are captured in the "Metadata" section (red arrow) and these are immutable by the user, but additional metadata may be added in the form of text, links, and images. The "Browse" button (blue arrow) will open the experiment for viewing in the EPIC-CoGe browser.

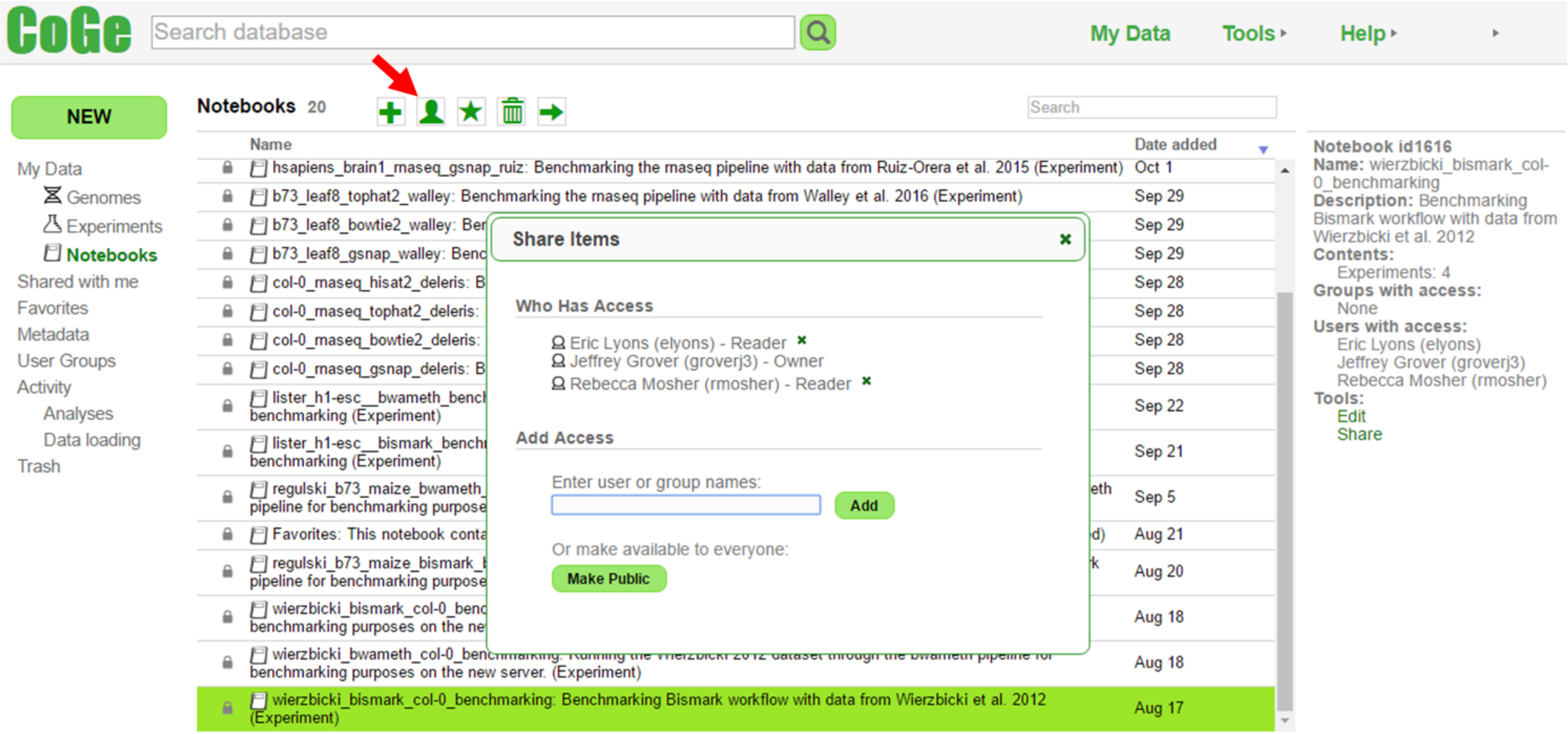

Figure S5. Sharing Data Within CoGe. Whole notebooks or individual experiment files can be shared with collaborators by clicking the “share” button on the right side of the My Data page or the button above the listing of notebooks (red arrow). Typing in a user name will allow searching through all of CoGe’s users. Additionally, data can be made public and viewable by all users from this area as well.

Data Size	Aligner	Source Data	Organism (Genome ID)	Run Time	Options
29,752,004 reads 1.66 GB (gzipped)	GSNAP	(Deleris *et al.*, 2012) (WT Col-0) SRR3709924	*Arabidopsis thaliana* (16911)	36 minutes, 37.57 seconds	Single-ended, phred33, CutAdapt: -q 25 -m 17<br>GSNAP: -N 1, -n 5, -Q –gap-mode none –nofails<br>Expression Analysis: enable, -q 20, -frag-bias-correct, -multi-read-correct
29,752,004 reads 1.66 GB (gzipped)	Bowtie2	(Deleris *et al.*, 2012) (WT Col-0) SRR3709924	*Arabidopsis thaliana* (16911)	1 hour, 58 minutes, 22.02 seconds	Single-ended, phred33, CutAdapt: -q 25 -m 17<br>Bowtie2: default options<br>Expression Analysis: enable, -q 20, -frag-bias-correct, -multi-read-correct
29,752,004 reads 1.66 GB (gzipped)	TopHat2	(Deleris *et al.*, 2012) (WT Col-0) SRR3709924	*Arabidopsis thaliana* (16911)	1 hour, 6 minutes, 58.08 seconds	Single-ended, phred33, CutAdapt: -q 25 -m 17<br>TopHat2: -g 1<br>Expression Analysis: enable, -q 20, -frag-bias-correct, -multi-read-correct
29,752,004 reads 1.66 GB (gzipped)	HiSat2	(Deleris *et al.*, 2012) (WT Col-0) SRR3709924	*Arabidopsis thaliana* (16911)	47 minutes, 33.30 seconds	Single-ended, phred33, CutAdapt: -q 25 -m 17<br>HISAT2: default options<br>Expression Analysis: enable, -q 20, -frag-bias-correct, -multi-read-correct
20,434,594 reads 1.74 GB (gzipped)	GSNAP	(Walley *et al.*, 2016) (*Zea mays* b73, leaf 8) SRR957442	*Zea mays* (25317)	45 minutes, 17.21 seconds	Single-ended, phred33, CutAdapt: -q 25 -m 17<br>GSNAP: -N 1, -n 5, -Q –gap-mode none –nofails<br>Expression Analysis: enable, -q 20, -frag-bias-correct, -multi-read-correct
20,434,594 reads 1.74 GB (gzipped)	Bowtie2	(Walley *et al.*, 2016) (*Zea mays* b73, leaf 8) SRR957442	*Zea mays* (25317)	1 hour, 29 minutes, 55.87 seconds	Single-ended, phred33, CutAdapt: -q 25 -m 17<br>Bowtie2: default options<br>Expression Analysis: enable, -q 20, -frag-bias-correct, -multi-read-correct
20,434,594 reads 1.74 GB (gzipped)	TopHat2	(Walley *et al.*, 2016) (*Zea mays* b73, leaf 8) SRR957442	*Zea mays* (25317)	1 hour, 15 minutes, 28.34 seconds	Single-ended, phred33, CutAdapt: -q 25 -m 17<br>TopHat2: -g 1<br>Expression Analysis: enable, -q 20, -frag-bias-correct, -multi-read-correct
20,434,594 reads 1.74 GB (gzipped)	HiSat2	(Walley *et al.*, 2016) (*Zea mays* b73, leaf 8) SRR957442	*Zea mays* (25317)	42 minutes, 59.59 seconds	Single-ended, phred33, CutAdapt: -q 25 -m 17<br>HISAT2: default options<br>Expression Analysis: enable, -q 20
73,730,964 reads 9.91 GB (gzipped)	GSNAP	(Ruiz-Orera *et al.*, 2015) (brain1) SRR2040575	*Homo sapiens* (25747)	3 hours, 22 minutes, 12.54 seconds	Paired end, phred33, CutAdapt: -q 25 -m 17<br>GSNAP: -N 1, -n 5, -Q –gap-mode none –nofails<br>Expression Analysis: enable, -q 20, -frag-bias-correct, -multi-read-correct
73,730,964 reads 9.91 GB (gzipped)	Bowtie2	(Ruiz-Orera *et al.*, 2015) (brain1) SRR2040575	*Homo sapiens* (25747)	14 hours, 13 minutes, 59.75 seconds	Paired end, phred33, CutAdapt: -q 25 -m 17<br>Bowtie2: default options<br>Expression Analysis: enable, -q 20, -frag-bias-correct, -multi-read-correct
73,730,964 reads 9.91 GB (gzipped)	TopHat2	(Ruiz-Orera *et al.*, 2015) (brain1) SRR2040575	*Homo sapiens* (25747)	11 hours, 35 minutes, 55.21 seconds	Single-ended, phred33, CutAdapt: -q 25 -m 17<br>TopHat2: -g 1<br>Expression Analysis: enable, -q 20, -frag-bias-correct, -multi-read-correct
73,730,964 reads 9.91 GB (gzipped)	HiSat2	(Ruiz-Orera *et al.*, 2015) (brain1) SRR2040575	*Homo sapiens* (25747)	6 hours, 25 minutes, 5.96 seconds	Paired end, phred33, CutAdapt: -q 25 -m 17<br>HISAT2: default options<br>Expression Analysis: enable, -q 20

Table S1. RNAseq Workflow Benchmarking Information. All aligners calculate FPKM with Cufflinks and read depth with SAMtools. All options selected are based on default values at the time of publication. Genome ID is assigned upon upload to CoGe and is searchable. Run times are dependent on server load and may vary.

Data Size	Aligner	Source Data	Organism (Genome ID)	Run Time	Options
93,770,828 reads 13.28 GB (gzipped)	GSNAP	(Sutinen *et al.*, 2014) Input: SRR923516 Rep 1: SRR1118386 Rep 2: SRR1118387	*Homo sapiens* (25747)	7 hours, 54 minutes, 43 seconds	Single-ended, phred33, CutAdapt: -q 25 -m 17 GSNAP: -N 1, -n 5, -Q –gap-mode none –nofails ChIP-seq Analysis: -size 250 -gsize 3000000000 -norm 10000000 -fdr 0.01 -F 3
93,770,828 reads 13.28 GB (gzipped)	Bowtie2	(Sutinen *et al.*, 2014) Input: SRR923516 Rep 1: SRR1118386 Rep 2: SRR1118387	*Homo sapiens* (25747)	14 hours, 10 minutes, 30 seconds	Single-ended, phred33, CutAdapt: -q 25 -m 17 Bowtie2: default options ChIP-seq Analysis: -size 250 -gsize 3000000000 -norm 10000000 -fdr 0.01 -F 3
93,770,828 reads 13.28 GB (gzipped)	TopHat	(Sutinen *et al.*, 2014) Input: SRR923516 Rep 1: SRR1118386 Rep 2: SRR1118387	*Homo sapiens* (25747)	28 hours, 21 minutes, 47 seconds	Single-ended, phred33, CutAdapt: -q 25 -m 17 TopHat2: -g 1 ChIP-seq Analysis: -size 250 -gsize 3000000000 -norm 10000000 -fdr 0.01 -F 3
93,770,828 reads 13.28 GB (gzipped)	HISAT2	(Sutinen *et al.*, 2014) Input: SRR923516 Rep 1: SRR1118386 Rep 2: SRR1118387	*Homo sapiens* (25747)	5 hours, 37 minutes, 43 seconds	Single-ended, phred33, CutAdapt: -q 25 -m 17 HISAT2: default options ChIP-seq Analysis: -size 250 -gsize 3000000000 -norm 10000000 -fdr 0.01 -F 3

Table S2. ChIPseq Analysis Benchmarking Information. All options selected are based on default values at the time of publication. Run times are dependent on server load and may vary.

Data Size	Analysis Tool	Source Data	Organism (Genome ID)	Run Time	Options
41,408,716 reads 2.05 GB (gzipped)	Bismark	(Wierzbicki *et al.*, 2012) (Col-0 ecotype) SRX156068	*Arabidopsis thaliana* (16911)	1 hour, 54 minutes, 59.73 seconds	Single-ended, phred33, Trim Galore: -q 20 --length 20, Bismark: -N 0 –L 20, Deduplicate, Minimum Coverage: 5
41,408,716 reads 2.05 GB (gzipped)	bwameth	(Wierzbicki *et al.*, 2012) (Col-0 ecotype) SRX156068	*Arabidopsis thaliana* (16911)	1 hour, 11 minutes, 0.45 seconds	Single-ended, phred33, Trim Galore: -q 20 --length 20, bwameth: Default options, Deduplicate, Minimum Coverage: 5
303,269,187 reads 24.99 GB (gzipped)	Bismark	(Regulski *et al.*, 2013) (b73 variety) SRX159573	*Zea mays* (25317)	30 hours, 59 minutes, 30.63 seconds	Paired end, phred33, Trim Galore: -q 20 --length 20, Bismark: -N 0 -L 20, Deduplicate, Minimum Coverage: 5
303,269,187 reads 24.99 GB (gzipped)	bwameth	(Regulski *et al.*, 2013) (b73 variety) SRX159573	*Zea mays* (25317)	53 hours, 56 minutes, 36.16 seconds	Paired end, phred33, Trim Galore: -q 20 --length 20, bwameth: Default options, Deduplicate, Minimum Coverage: 5
570,423,799 reads 43.12 GB (gzipped)	Bismark	(Lister *et al.*, 2009) (H1 embryonic stem cells) SRX006239	*Homo sapiens* (25747)	42 hours, 19 minutes, 13.70 seconds	Single-ended, phred33, Trim Galore: -q 20 --length 20, Bismark: -N 0 -L 20, Deduplicate, Minimum Coverage: 5
570,423,799 reads 43.12 GB (gzipped)	bwameth	(Lister *et al.*, 2009) (H1 embryonic stem cells) SRX006239	*Homo sapiens* (25747)	22 hours, 15 minutes, 55.87 seconds	Single-ended, phred33, Trim Galore: -q 20 --length 20, bwameth: Default options, Deduplicate, Minimum Coverage: 5

Table S3. Bisulfite Sequencing Workflow Benchmarking Information. Bismark runs in Bowtie2 mode. All options selected are based on default values at the time of publication with the exception of deduplication. Run times are dependent on server load and may vary.

References

Deleris,A. *et al.* (2012) Loss of the DNA Methyltransferase MET1 Induces H3K9 Hypermethylation at PcG Target Genes and Redistribution of H3K27 Trimethylation to Transposons in Arabidopsis thaliana. *PLoS Genet.*, **8**.

Heinz,S. *et al.* (2010) Simple Combinations of Lineage-Determining Transcription Factors Prime cis-Regulatory Elements Required for Macrophage and B Cell Identities. *Mol. Cell*, **38**, 576–589.

Kim,D. *et al.* (2015) HISAT: a fast spliced aligner with low memory requirements. *Nat. Methods*, **12**, 357–360.

Kim,D. *et al.* (2013) TopHat2: accurate alignment of transcriptomes in the presence of insertions, deletions and gene fusions. *Genome Biol.*, **14**, R36.

Krueger,F. and Andrews,S.R. (2011) Bismark: a flexible aligner and methylation caller for Bisulfite-Seq applications. *Bioinformatics*, **27**, 1571–1572.

Langmead,B. *et al.* (2009) Ultrafast and memory-efficient alignment of short DNA sequences to the human genome. *Genome Biol.*, **10**, R25.

Li,H. *et al.* (2009) The Sequence Alignment/Map format and SAMtools. *Bioinformatics*, **25**, 2078–2079.

Lister,R. *et al.* (2009) Human DNA methylomes at base resolution show widespread epigenomic differences. *Nature*, **462**, 315–322.

Martin,M. (2011) Cutadapt removes adapter sequences from high-throughput sequencing reads. *EMBnet.journal*, **17**, 10.

McKenna, A. *et al.* (2010) The Genome Analysis Toolkit: a MapReduce framework for analyzing next-generation DNA sequencing data. *Genome Res.,* **20,** 1297-1303.

Pedersen,B.S. *et al.* (2014) Fast and accurate alignment of long bisulfite-seq reads. *arXiv Prepr.*, 1–2.

Regulski,M. *et al.* (2013) The maize methylome influences mRNA splice sites and reveals widespread paramutation-like switches guided by small RNA. *Genome Res.*, **23**, 1651–1662.

Rimmer,A. *et al.* (2014) Integrating mapping-, assembly- and haplotype-based approaches for calling variants in clinical sequencing applications. *Nat. Genet.*, **46**, 912–918.

Ruiz-Orera,J. *et al.* (2015) Origins of De Novo Genes in Human and Chimpanzee. *PLoS Genet.*, **11**, 1–24.

Sutinen,P. *et al.* (2014) SUMOylation modulates the transcriptional activity of androgen receptor in a target gene and pathway selective manner. *Nucleic Acids Res.*, **42**, 8310–8319.

Trapnell,C. *et al.* (2010) Transcript assembly and quantification by RNA-Seq reveals unannotated transcripts and isoform switching during cell differentiation. *Nat. Biotechnol.*, **28**, 511–515.

Walley,J.W. *et al.* (2016) Integration of omic networks in a developmental atlas of maize. *Science (80-.).*, **353**, 814–818.

Wierzbicki,A.T. *et al.* (2012) Spatial and functional relationships among Pol V-associated loci, Pol IV-dependent siRNAs, and cytosine methylation ir the Arabidopsis epigenome. *Genes Dev.*, **26**, 1825–1836.

Wu,T.D. and Nacu,S. (2010) Fast and SNP-tolerant detection of complex variants and splicing in short reads. *Bioinformatics*, **26**, 873–881.
