# Supplementary material for: CoGe LoadExp+: A web‐based suite that integrates next‐generation sequencing data analysis workflows and visualization
Source: Plant Direct. 2017 Jul 20;1(2):10.1002/pld3.8. doi: 10.1002/pld3.8 (PMC6508507; doi:10.1002/pld3.8)
Supplement: Supplementary file 1 [file PLD3-1-0-s001.pdf]

We would like to thank the reviewer and editor for their comments as they have help us improve the paper. Please find our specific responses and detailed changes the manuscripts below. Review and editor comments are in black; our responses are in blue.

The editor's comments:
A minor revision is required. Some other relevant platforms, such as QuickNGS and MAV-seq, besides Galaxy, have been developed for NGS data analysis and integration. Authors can review these platforms and make a comparison with CoGe LoadExp+.

Thank you for this suggestion. We have updated the text to include more explicit comparison to existing tools besides galaxy. Unfortunately, we had problems getting information about MAV-seq. There is a publication in Nature Methods (online only) and a published abstract from "Human genome meeting 2016" that detail it as having essentially every feature possible.

The Jackson Laboratory's website about it contains no information about where to find it to use online or its source code. Perhaps it's an internal tool for them only? Or it's still under development. https://www.jax.org/research-and-faculty/tools/mav-seq

To emphasize the public access and open availability of LoadExp+, we:

- Line 32 of abstract: added "using a publicly available web-based platform"
- Line 42: added "on a publicly available web-accessible server"
- Line 188-193: updated text "With the recent increased accessibility of sequencing-based analyses, several NGS workflow management tools have become available to bridge the gap between bioinformatics expertise and researchers who want to analyze data quickly and efficiently. LoadExp+ distinguishes itself from similar tools due to its integration with CoGe's thousands of available genomes, a modern and feature-rich genome browser (EPIC-CoGe), and easily accessible workflows for numerous genomic and epigenomic analyses on a public server."
- Lines 198-209: added "Other NGS workflow management platforms such as QuickNGS [18] or Chipster [19] lack public servers accessible to all users, requiring labs or institutes to maintain an installation themselves. Available web-based genomics resources and workflow management tools, in particular, underserve users that wish to analyze bisulfite sequencing. Web-based tools for bisulfite sequencing such as WBSA [20] exist, and are publicly available, but lack the integration with other NGS workflows that is present in LoadExp+."
- Lines 223-225: added "The combination of LoadExp+'s workflows with EPIC-CoGe's visualizations and data manipulation functions provide LoadExp+ with unique capabilities compared to other NGS workflow applications."

Reviewer #1 :
I have two minor suggestions:

1. please discuss in more details the pros/cons of LoadExp+ vs. Galaxy. The current discussion implicates that galaxy can have the functions of loadexp+ (if installed and configured by a skilled bioinformatician). Are there any functions that loadexp+ absolutely has but galaxy does not?

Please see the response to the editor’s comment. Also, we have re-emphasized in the text those options which are specific to LoadExp+:

- Automatic integration with our JBrowse-based genome browser (EPIC-CoGe).
  - This allows real-time manipulation and analysis of data tracks to find minima/maxima or data that overlaps annotated genomic features of interest.
  - It also enables users to rescale and normalize data on the fly.
- Mixing and matching visualization of both public and private data at the same time.
- Users can make their data public, keep it completely private, or share it with a set of collaborators
- Analyses can be performed using any of the genomes available in CoGe, or users can upload their own.
- All analysis workflows supported by LoadExp+ are usable through a public server.

2. it's not very clear where to find loadexp+ on the current CoGe website. The website has a Load Experiment tool, and I assume it is loadexp+. Click on the Documentation link underneath opens the doc page called FlowGe. Please make corrections to use just one term if they are referring to the same tool (not to confuse readers and potential users).

Our apology for this confusion. We had tried different names for LoadExp+. FlowGe was one that we originally used, but users found the name to be non-informative. Our apologies for not updating the wiki page appropriately as well as having confusing text in CoGe. The following have been changed:

- The CoGePedia wiki page has been changed to “LoadExp+”: https://genomevolution.org/wiki/index.php/LoadExp%2B
  - This includes having redirects from the old wiki pages “FlowGe” and “LoadExperiment”.
- The text on CoGe’s homepage has been changed from “Load Experiment” to “Load Experiment (LoadExp+)”
- The “Tools” menu bar has been changed from “Load Experiment” to “LoadExp+”
- Under the “My Data” page (User.pl), the “New” dropdown menu has been changed from “New Experiment” to “New Experiment (LoadExp+)
- The LoadExp+ webpage has been changed:
  - Popup title changed from “LoadExperiment” to “LoadExp+”
  - Popup text changed from “Welcome to LoadExperiment!” to “Welcome to LoadExp+!”
  - Help links have been updated to go to correct wiki page: https://genomevolution.org/wiki/index.php/LoadExp%2B
  - Main page’s help link points to correct wiki page: https://genomevolution.org/wiki/index.php/LoadExp%2B

- Links in the wiki have been updated to point to the correct wiki page on the following pages:
    - ChIPseq annotation
    - GATK annotation
    - Cufflinks annotation
    - Alignment annotation
    - CoGeSNPs annotation
    - Samtools annotation
    - Platypus annotation
    - Ensembl GTF annotation
- In GenomeInfo (the page for viewing a genome in CoGe), the “Load Experiment” button was updated to “LoadExp+”
